# Role of Inflammasomes in Neuroimmune and Neurodegenerative Diseases: A Systematic Review

**DOI:** 10.1155/2018/1549549

**Published:** 2018-04-17

**Authors:** Yue Lang, Fengna Chu, Donghui Shen, Weiguanliu Zhang, Chao Zheng, Jie Zhu, Li Cui

**Affiliations:** ^1^Department of Neurology and Neuroscience Center, First Hospital of Jilin University, Changchun, Jilin Province, China; ^2^Department of Neurobiology, Care Sciences & Society, Division of Neurodegerneration, Karolinska Institutet, Karolinska University Hospital Huddinge, Stockholm, Sweden

## Abstract

Inflammasomes are multiprotein complexes that can sense pathogen-associated molecular patterns and damage-associated molecular signals. They are involved in the initiation and development of inflammation via activation of IL-1*β* and IL-18. Many recent studies suggest a strong correlation between inflammasomes and neurological diseases, such as multiple sclerosis (MS), Alzheimer's disease (AD), and Parkinson's disease (PD). Several components of inflammasomes, such as nucleotide-binding oligomerization domain- (NOD-) like receptor, absent in melanoma 2- (AIM2-) like receptors (ALRs), apoptosis-associated speck-like protein containing a caspase recruitment domain (ASC), and caspase-1, as well as the upstream factors and downstream effectors, are associated with the initiation and development of MS and its animal model, experimental autoimmune encephalomyelitis. Additionally, inflammasomes affect the efficacy of interferon-*β* therapy in patients with MS. Finally, the strong association of inflammasomes with AD and PD needs to be further studied. In this review of latest literatures, we comprehensively tease out diverse roles of different kinds of inflammasomes in neuroimmune and neurodegenerative diseases, especially in the perspective of double roles involved in pathogenesis, and identify future research priorities.

## 1. Introduction

Inflammasomes are multiprotein complexes with an inherent ability to elicit innate immune responses by sensing damage signals and microbial attack [1]. Inflammasomes exist in the cytosol of several types of cells, including immune cells (such as T cells, B cells, dendritic cells, and macrophages), neural cells [2], microglia [3], and astrocytes [4] as well as pulmonary endothelial cells [5]. Inflammasomes play a critical role in the development of neurological, immune, and neurodegenerative diseases, such as multiple sclerosis (MS), Alzheimer's disease (AD), and Parkinson's disease (PD). The multimeric complexes in inflammasomes have classically been referred to as “damage sensors,” since nucleotide-binding oligomerization domain- (NOD-) and absent in melanoma 2- (AIM2-) like receptors recognize and interact with pathogen-associated molecular patterns, which in turn trigger a series of immune reactions. Recently, there has been an increase in the research on inflammasomes. Several studies have documented the increased inflammasome components and inducing factors, such as adenosine triphosphate (ATP) and uric acid, during the development of MS and experimental autoimmune encephalomyelitis (EAE), an animal model of MS. In this work, we provide a comprehensive overview of the role of inflammasomes in neuroimmune and neurodegenerative diseases based on a review of contemporary literature.

## 2. Structure and Function of Inflammasomes

Inflammasomes contain three components: sensors, apoptosis-associated speck-like protein containing a caspase recruitment domain (ASC), and an enzymatic component. Based on the structural features, sensors can be classified into three types: NOD-like receptors (NLRs), AIM2-like receptors (ALRs), and pyrin. The sensors have the ability to detect pathogen-associated molecular patterns (PAMPs) [6] or damage-associated molecular patterns (DAMPs) and cytosolic double-stranded DNA [7]. Detection of these stimuli triggers the assembly of the three components based on the nucleating ability of the pyrin domain (PYD) and the caspase recruitment domain (CARD) [8, 9]. The assembled complexes act as proteolytic cleavers which activate the precursor of interleukin-1*β* (IL-1*β*) and IL-18, which are involved in a series of immune and inflammatory processes [10–12]. The NLR family, pyrin domain-containing 1 [13], 3 [14], 4 [15], and 12 [16] (NLRP1, NLRP3, NLRP4, and NLRP12, resp.), are NLRs that have been shown to be involved in inflammasome assembly, while AIM2 is the most well-characterized ALR. AIM2 detects DNA through a HIN domain [17], while NLRP3 [18] detects intracellular DNA by the stimulator of IFN genes- (STING-) mediated DNA-sensing pathway [18]. In general, IL-1*β* is cleaved by caspase-1. However, some previous studies also indicated that IL-1*β* can be processed by caspase-8 [19] or caspase-11 through a noncanonical inflammasome [20].

Most inflammasomes promote inflammation by inducing production of inflammatory factors IL-1*β* and IL-18. However, some inflammasomes also have anti-inflammatory effects. The inhibitory effect of NLRX1 on microglial-induced inflammation in EAE was first reported by Eitas et al. [21]. Moreover, inflammasomes are capable of maintaining the balance of the gut microflora and prevent enteritis from progressing to a tumor [22].

## 3. Factors Involved in Inflammasome Activation and Regulation

Since inflammasomes lower the threshold of immune response [23], the search for the factors involved in their activation and regulation aiming at identifying therapeutic targets against autoimmune disease has evoked much interest. The initiation of inflammasomes is not well characterized; however, the following mechanism of activation of NLRP3 has been described: First, the bacterial or viral RNA [24], ATP [25], uric acid crystals [26], silica [27], and other similar factors interact with toll-like receptors (TLRs), NOD2, TNFR1, or TNFR2 to induce a cellular expression of NLRP3. Second, PAMP or DAMP triggers NLRP3 to initiate inflammasome formation. In the first stage, P2X7/P2X4 has been shown to be a receptor of ATP [28], whereas pannexin 1 (Panx1) mediates the release of ATP from cells [29]. P2X(4) receptors are another important signal that activates inflammasomes. It has been reported that P2X(4) influences NLRP1 inflammasome signaling in spinal cord injury [30] and osteoarthritis [31]. Moreover, biglycan, a kind of leucine-rich repeat proteoglycan, can also activate NLRP3 inflammasomes through interaction with TLR2/4 and purine P2X4/P2X7 receptors on macrophages [32]. Antiapoptotic proteins, activated T cells, and microRNAs are negative regulators of inflammasomes [33]. Tumor necrosis factor (TNF) *α*-induced protein 3 (TNFAIP3) and the binding of antiapoptotic proteins Bcl-2 and Bcl-X(L) have been shown to dampen the NLRP1 inflammasome [34, 35]. NF-*κ*B is also one of the activation signals for inflammasomes [36]. Atorvastatin was shown to suppress inflammasomes in monocytes via the TLR4/MyD88/NF-*κ*B pathway [37]. The myeloid-specific microRNAs, miR-223 and miR-7, suppress NLRP3 inflammasome activity via inhibition of NLRP3 protein expression [38, 39]. Mouse effector and memory CD4^+^ T cells were shown to suppress inflammasomes of macrophages via cell-to-cell contact [40]. Human CD4^+^ memory T cells suppress NLRP3 activation by downregulating P2X7R signaling [41]. Furthermore, it has been reported that caspase-12 associated with caspase-1 inhibits its activity [42]. In addition, polyoxotungstate-1 (POM-1) can inhibit the events related with ATP-dependent inflammasome activation [43]. Probenecid, a Panx1 inhibitor, protects against oxygen-glucose deprivation injury in primary astrocytes by regulating inflammasome activity [44]. Moreover, one interesting finding is that in primary macrophage cultures, low intracellular K(+) and the membrane channel Panx1 induce inflammasome activation, while in the primary neuron and astrocyte cultures, high extracellular potassium opens Panx1 channels leading to caspase-1 and inflammasome activation [45]. The reason for this discrepancy is still unclear; it is surmised that there might be an unknown factor suppressing caspase-1. Factors that negatively regulate inflammasome activity are shown in Table 1.

## 4. Inflammasomes in MS/EAE

MS is an immune-mediated, chronic inflammatory demyelinating disease of the central nervous system (CNS). The hallmark of MS is recurrent neurological dysfunction and a progressive disease course. EAE is an animal model of MS [46] that is characterized by mononuclear cell infiltration around small vessels and demyelination in CNS mediated by specific sensitized CD4^+^ T cells.

Dumas et al. [47] demonstrated that the pertussis toxin could promote the formation of a pyrin-dependent inflammasome in EAE. Elevated levels of IL-1*β* have also been reported in the cerebrospinal fluid (CSF) of MS patients before clinical relapse, and caspase-1 expression is detected in MS plaques [48].

EAE mice with *NLRP3* gene knockout experienced a different disease course. The NLRP3^−/−^ mice had a significantly delayed disease course and less severe disease [49]. Subsequently, the higher dose of immunizing agents in ASC^−/−^ and NLRP3^−/−^ mice implies that inflammasomes are involved in the progression of EAE. Additionally, the amount of inflammasome-associated protein mRNA, such as in NLRP1, caspase-1, caspase-3, ASC, and pro-IL-1*β*, were shown to be elevated 2 weeks after injection of myelin oligodendrocyte glycoprotein peptide [13, 50–53]. In a chlorpromazine- (CPZ-) induced demyelination mouse model, the progesterone treatment group exhibited a decrease in neurological behavioral deficit scores accompanied by decreased levels of NLRP3 inflammasomes [54]. This research suggests that NLRP3 inflammasomes are involved in the pathogenesis of the CPZ-induced demyelination mouse model. Moreover, the occurrence of MS-like symptoms in patients with other autoimmune diseases and inflammasome-associated genetic mutations indicates that inflammasomes are involved in the pathogenesis of MS. For example, mutations of the pyrin domain of Mediterranean fever gene (MEFV gene) have been linked to a higher susceptibility to more progressive or severe MS [55–57]. Patients with familial Mediterranean fever who have CAPS-associated V198M and Q703K mutations tend to experience MS (with comorbidity reaching as high as 53%) [58]. Magnetic resonance imaging of the brain of one patient with Muckle-Wells syndrome showed MS-like pathology [59, 60].

However, not all NLRP proteins promote inflammation. Anti-inflammatory actions of NLRX1 have been demonstrated in EAE, and NLRX1^−/−^ mice were shown to display higher clinical scores than wild-type controls were [21]. Additionally, NLRP12 also inhibits the nuclear factor-*κ*B (NF-*κ*B) pathway by interacting with NF-*κ*B-inducing kinase and the TNF receptor-associated factor (TRAF) 3 in innate immune cells without inflammasome formation [61–63]. Inflammatory response in the NLRP12^−/−^ mouse EAE model is much stronger than that in the control group [64].

## 5. The Role of Inflammasomes in the Pathogenesis of MS/EAE

Inflammasomes promote activation of both IL-1*β* and IL-18 and migration of Th1 and Th17 cells into CNS. Although there is no consensus as to whether pyrin promotes [65] or inhibits [66] the function of inflammasomes, the inflammasomes are believed to play a critical role in neuroimmune diseases.

IL-1*β* and IL-18 are the effectors of inflammasomes. IL-1*β* has been detected in white and gray matter lesions in a mouse EAE model [67]. In this model, IL-1*β* is secreted by infiltrating monocytes [68] and meningeal mast cells [69], while in a rhesus EAE model, IL-1*β* is mainly induced in the CNS itself [70]. Since IL-1*β* is associated with impairment of the blood-brain barrier and the blood-spinal cord barrier [71, 72], it is believed to promote immune cell migration into the CNS, which is a crucial link to EAE. Moreover, IL-1*β* is known to affect T cell responses [73]. It is secreted by mast cells and induces the expression of the granulocyte-macrophage colony-stimulating factor (GM-CSF), which is an important factor in T cell encephalitogenicity [74]. Besides, IL-1*β* induces the differentiation of Th17 cells [75] and subsequently aggravates EAE [76]. IL-18 was shown to promote autoimmunity by stimulating innate IL-17 production by T cells [77], and the increased levels of serum IL-18 in patients with MS illustrate its key role in the pathogenesis of MS [78].

Th1 and Th17 cells are critical to the progress of EAE [79], and the inflammasomes were shown to induce EAE via modulation of their autoimmune response. Gris et al. [49] demonstrated that NLRP3 could possibly impair Th1 and Th17 responses by affecting caspase-1-dependent cytokines, and hence, NLRP3 could induce EAE. However, there are conflicting views pertaining to the role of Th17 in the NLRP3^−/−^ and ASC^−/−^ mouse EAE models. Inoue et al. [80] reported that reduction in Th17 is not crucial for a reduced clinical score in the NLRP3^−/−^ and ASC^−/−^ mouse EAE models.

Moreover, inflammasomes could also enhance EAE by affecting Th1 and Th17 immigration to the CNS. Inoue et al. reported that NLRP3 inflammasomes do not induce an increase in the population of Th17 cells; instead, they contribute to T cell chemotactic activity in the CNS [80]. During the process, CD4^+^ T cells are activated by IL-1*β* and IL-18 produced by antigen-presenting cells (dendritic cells and macrophages), which have copious amounts of inflammasomes; as a consequence, there is an increase in the expression levels of chemotaxis-related proteins (such as osteopontin, CCR2, and CXCR6) [80].

In addition, inflammasomes are the cofactor of some substances that are involved in EAE pathogenesis, such as integrin-associated protein (IAP) CD47 and Panx1. *In vivo* administration of exogenous IL-1*β* has been shown to promote the infiltration of CD47^−/−^ Th17 cells into the CNS. Mechanically, blocking of CD47 activates Src, subsequently inducing and then decreasing the degradation level of inducible nitric oxide synthase. As a result, the level of nitric oxide (NO) increases and suppresses inflammasome activation-induced IL-1*β* production. As mentioned previously, lower IL-1*β* reduces the expressions of IL-1R and migration-related chemokine receptors on Th17 cells, thereby suppressing EAE development [81]. The reason why the plasma membrane channel Panx1 could contribute to EAE progression is that Panx1 mediates ATP release and further triggers inflammasome activation [82].

**Figure 1:**
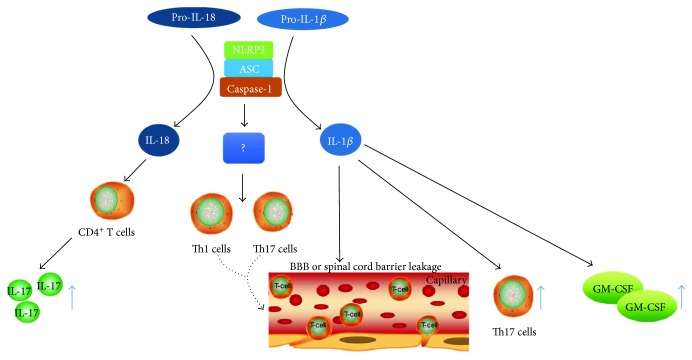
Inflammasomes and factors involved in the pathogenesis of MS/EAE. NLRP3 inflammasomes convert pro-IL-1*β* and pro-IL-18 to IL-1*β* and IL-18. IL-18 stimulates CD4^+^ T cells to produce IL-17; NLRP3 inflammasomes contribute to T cell chemotactic activity in the CNS; IL-1*β* induces the differentiation of Th17 cells and promotes them infiltrate into the CNS; IL-1*β* also induces the production of GM-CSF, which is an important factor in T cell encephalitogenicity. IL-1*β*: interleukin-1*β*; IL-18: interleukin-18; IL-17: interleukin-17; NLRP3: NOD-like receptor pyrin domain-containing 3; ASC, apoptosis-associated speck-like protein containing a caspase recruitment domain; BBB: blood-brain barrier; GM-CSF: granulocyte-macrophage colony-stimulating factor.

Interestingly, inflammasomes have been evidenced to excrete exosomes acting on nearby macrophages and activating the NF-*κ*B signal pathway, which may enhance the immune response in autoimmune diseases [83]. These findings regarding the mechanism of inflammasomes involved in inflammation may give new insights into the pathogenesis of MS/EAE. The inflammasomes and factors involved in MS/EAE are shown in Figure 1.

## 6. Inflammasomes and the Role of IFN*γ* and IFN*β* in EAE

According to Inoue et al., EAE can be of two types: type A which is *NLRP3*-dependent and type B, which is *NLRP3*-independent and resistant to interferon-*β* (IFN*β*) treatment [84]. Type B EAE accounts for one-third of all EAE animals [85]. The two subtypes are amenable to alteration during EAE development.

IFN*β* is effective in relapsing-remitting multiple sclerosis (RRMS) via inhibition of *NLRP3* [84, 86]. Type I interferon was shown to inhibit NLRP1/NLRP3 inflammasome and then reduce the expression levels of pro-IL-1*β*/pro-IL-18 [86]. Besides, binding of IFN*β* with IFN receptor I on antigen-presenting cells (APCs) inhibits suppression of cytokine signaling (SOCS), which can downregulate a member of the Rho family of GTPases, Rac1-guanosine triphosphate (Rac1-GTP), through SOCS-1 and induce generation of reactive oxygen species (ROS) [84]. The increase in ROS level is conducive to activation of inflammasomes. However, these mechanisms do not work on NLRC4 inflammasomes. Moreover, there is proof of involvement of the other inflammasome components in the pathogenesis of MS: NOD2 gene polymorphism rs5743291 was shown to activate Th2 and Th17 cells in patients with MS [87]. Further, targeting of NOD2 pathways with a NOD2-activating agent, MIS416, was shown to modulate the Th response and alleviate EAE [88].

In a study by Noroozi et al., MS patients treated with IFN*β* experienced a decrease in plasma IL-1*β* levels as well as the expressions of NLRP3, NLRC4, and AIM2 in peripheral blood mononuclear cells [89]. Similar results have been reported from a study by Malhotra et al.: MS patients who responded well to IFN*β* showed lower mRNA levels of NLRP3 and IL-1*β* as compared to their pretreatment levels; conversely, nonresponders experienced upregulation of NLRP3 and IL-1*β* mRNA levels after three months of treatment with IFN*β* [90]. The underlying mechanism of resistance of the NLRP3 inflammasome-independent pattern to IFN*β* treatment is not completely understood. However, Inoue et al. [91] have shown that membrane-bound lymphotoxin and the chemokine receptor CXCR are involved in this process and antagonizing these receptors ameliorates *NLRP3*-independent EAE.

## 7. The Targets of Treatment in MS/EAE

Owing to the key role of inflammasomes in the development of EAE or MS, research on the means to inhibit their function is of much clinical relevance. The upstream factors and downstream effectors of inflammasomes are attractive therapeutic targets in the context of EAE/MS. JC-171, a hydroxyl sulfonamide analogue, which is a selective NLRP3 inflammasome inhibitor, has been shown to exhibit preventive and therapeutic effects in the EAE setting [92]. In a study by Coll et al. [93], MCC950 (a diarylsulfonylurea-containing compound that inhibits IL-1*β*) was shown to specifically inhibit canonical and noncanonical NLRP3 activation in EAE. Moreover, as Gao et al. [81] described that CD47-Fc fusion protein, the inhibitor of CD47, which is capable of inducing NO production that suppresses inflammasome activation-induced IL-1*β* expression, could also prevent and ameliorate EAE. Similarly, cladribine, an adenosine deaminase inhibitor, was shown to suppress excitatory postsynaptic currents induced by IL-1*β*, which has been proven to be one of the mechanisms for treatment of MS [94]. Furthermore, NLRP3 is involved in mediating the therapeutic effect of prednisone [95], cannabinoid receptor 2 (CB2R) [96], and periodontal ligament stem cells [97] in EAE.

## 8. Inflammasomes in Alzheimer's Disease

Alzheimer's disease (AD) is a neurodegenerative disease characterized by progressive cognitive impairment. It accounts for 50% to 70% of cases with dementia in the elderly [98] and is viewed as one of the great health-care challenges of the 21st century [99]. However, there is no effective cure to halt the progress of this disease [99]. AD is pathologically characterized by A*β* deposit. The A*β* deposit and damage-associated molecular pattern molecules (DAMPs) released by the subsequent neuronal injury are sensed by inflammasomes, which initiates an innate immune response [100, 101]. The messenger RNA (mRNA) levels of the NLRP1 and NLRP3 components increase in AD [102]. NLRP1 induces caspase-1 and caspase-6, and this pathway is involved in the progression of AD [103]. In addition to exhibiting lower caspase-1 and IL-1*β* activity levels and enhanced A*β* clearance, NLRP3^−/−^ mice display better memory than do NLRP3 wild-type mice. Moreover, knocking down NLRP3 promotes M1-type microglial bias to the M2 type. This helps in A*β* clearance and tissue remodeling in the APP/PS1 model as M2-type microglia are efficient at phagocytosis [104]. The mechanism of activation of NLRP3 is yet to be clarified; however, in microglia, NLRP3 inflammasomes were shown to be activated not only by A*β* via the TLR4-MyD88-NF-*κ*B pathway in microglia but also by P2X7R as in the EAE model and cathepsin B (CTSB). In addition, NLRP10, another NOD-like receptor, has been shown to attenuate A*β*-induced caspase-1 activation and IL-1*β* release [105].

**Figure 2:**
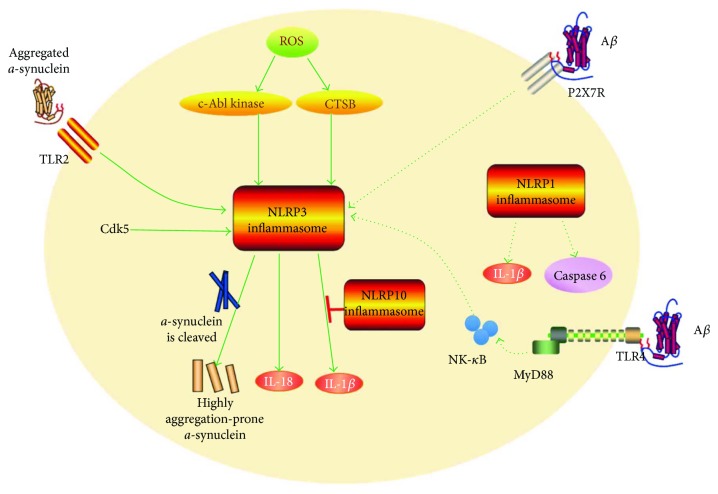
Schematic illustration of the role of inflammasomes in Parkinson's disease and Alzheimer's disease. NLRP1 induces IL-1*β* production and caspase 6 activation, which are factors involved in the progress of AD. A*β* activates NLRP3 inflammasomes via the P2X7R receptor or TLR4-MyD88-NF-*κ*B pathway, *α*-synuclein activates the toll-like receptor 2 (TLR2) which promotes the assembly of NLRP3 and induces IL-1*β* synthesis, ROS activates NLRP3 through c-Abl kinase or upregulates CTSB activity, Cdk5 could activate NLRP3 inflammasome, caspase-1 could cleave *α*-synuclein into a highly aggregation-prone specie, and NLRP10 attenuates A*β*-induced caspase 1 activation and IL-1*β* release. PD: Parkinson's disease; AD: Alzheimer's disease; TLR2: toll-like receptor 2; IL-1*β*: interleukin-1*β*; CTSB: cathepsin B; Cdk5: cyclin-dependent kinase 5; ROS: reactive oxygen species.

The fact that the overexpression of the effectors of inflammasomes, IL-1*β* and IL-18, initiates the inflammatory process in AD patients verifies the association between inflammasome and AD [106, 107]. Kitazawa et al. demonstrated that the IL-1*β* signal cascade is an important pathogenic factor of AD; its blockade was shown to ameliorate pathological changes in a mouse model of AD [108]. In a study by Craft et al., knocking out the IL-1*β* receptor antagonist in a mouse model of AD was shown to aggravate the neuropathological sequelae [109]. The above-mentioned investigations indicate that inhibition of inflammasome activation might be a potential therapeutic target for AD. The inflammasomes involved in AD are shown in Figure 2.

## 9. Inflammasomes in Parkinson's Disease

Parkinson's disease (or paralysis agitans) is a common neurodegenerative disorder, which is characterized by loss of dopaminergic neurons [110] and aggregation of the *α*-synuclein deposit [111]. Activation of inflammasomes and elevation of serum caspase-1 and IL-1*β* levels have been demonstrated in nigrostriatal DA regions of the PD mouse model as well as in the brain and cerebrospinal fluid of PD patients [112–114]. Inflammasomes could be activated by oxidative stress and excessive activated microglia, both of which play an important role in the pathogenesis of PD. To be specific, oxidative stress from ROS activates NLRP3 through c-Abl kinase or upregulates CTSB activity in microglial cells [115, 116]. Additionally, cyclin-dependent kinase 5 (Cdk5) is also an essential factor for activation of inflammasomes in neurons [114]. Caspase-1- and caspase-3-related apoptotic cell death is a crucial link, through which epidemiological risk factors such as MPP (+), paraquat, dieldrin, and salsolinol can cause PD [117, 118]. Furthermore, caspase-1 could directly cleave *α*-synuclein into a highly aggregation-prone variant, which subsequently forms aggregated *α*-synuclein and assaults neurons [119]. Noteworthily, despite of the correlation between caspase-1 and PD, caspase-1 may not be the determining factor of dopaminergic neuronal death in vitro, or the efficacy of caspase-1 inhibitors may depend on the extent of apoptotic stress [120]. Specifically, caspase-1 inhibitors did not improve the survival of grafted dopaminergic neurons in 1-methyl-4-phenyl-1,2,3,6-tetrahydropyridine- (MPTP-) induced PD models [120], and caspase-1 inhibitors could not prevent dopaminergic neuronal death in MPTP parkinsonian mice [121].

The function of NLRP3 and caspase-1 is closely related to *α*-synuclein, the pathogenic factor of PD. *α*-Synuclein activates the toll-like receptor 2 (TLR2) that promotes the assembly of NLRP3 [122] and induces synthesis of IL-1*β* [123]. The increase in IL-1*β* expression level induces production of ROS and release of CTSB, which in turn activates NLRP3 [115, 123]. Besides, *α*-synuclein was shown to promote both TLR4/NF-*κ*B and NLRP3/caspase-1 signals in adult neural stem cells (ANSCs), and both NLRP3 knockdown and caspase-1 deficiency reverse the antiproliferation effect of *α*-synuclein on ANSCs [39]. In terms of the treatment of PD, pharmacological inhibitions for inflammasome activation-related molecules, such as caspase-1, microRNA-7, CTSB, c-Abl, and Cdk5, may open up novel therapeutic avenues. The inflammasomes involved in PD are shown in Figure 2.

## 10. Closing Remarks

Inflammasomes are supramolecular signaling complexes involved in the inflammatory process. They consist of three components: sensors, ASC, and caspases. There are subvariants of sensors and caspases, such as NLRP1, NLRP3, caspase-1, and caspase-4, marking variable inflammasomes with different biological functions. Most of the inflammasomes contribute to the development of neuroimmune and neurodegenerative diseases such as the NLRP3 inflammasome, while some have anti-inflammatory properties such as NLRX1 inflammasome and NLRP12 inflammasome. In the pathogenesis of MS/EAE, inflammasomes promote Th1 and Th17 cells migrating into CNS. Furthermore, they enhance the immune response in EAE by secreting exosomes to act on nearby macrophages. In patients and animal models of AD and PD, the component of inflammasomes, such as caspase-1, and the effector IL-1*β* and IL-18, all can aggravate the A*β*- or *α*-synuclein-induced pathological process. Though substantial evidence has proved the vital roles of inflammasomes, the exact mechanism underlying inflammatory reactions in these disorders is yet to be fully understood. Theoretically, inflammasome antagonists may exhibit a protective effect on the patients with neuroimmune or neurodegenerative disorders via multiple pathways. Nevertheless, the efficacy of inflammasome-targeted therapies still needs further investigations. In addition, the exact pathogenesis of *NLRP3*-independent MS is still unclear.

Finally, the most reported inflammasomes involved in the pathogenesis of neuroimmune and neurodegenerative diseases are NLRP3 and NLRP1; whether other inflammasomes are potential important factors contributing to these disorders is worth studying in the future.

## Figures and Tables

**Table 1:** The negative regulation factors of inflammasomes.

Regulation of factors	Target	Mechanism
A20/TNFAIP3 [34]	NLRP1	Binding with Bcl-2 and Bcl-X(L)
miR-223 [38]	NLRP3	Acting on a conserved binding site within 3′ untranslated region of NLRP3
miR-7 [39]	NLRP3	Posttranscriptionally controlled NLRP3 expression
Human CD4^+^ memory T cells [41]	NLRP3	Down-regulation of P2X7R signaling molecules
Caspase-12 [42]	Caspase-1	Associated with caspase-1
POM-1 [43]	NLRP1	Inhibit the events related with ATP-dependent inflammasome
Probenecid [44]	NLRP3	Block pannexin 1 channel and high extracellular potassium
Type I interferons [86]	NLRP1/NLRP3	Via the STAT1 transcription factor

A20/TNFAIP3: tumor necrosis factor *α*-induced protein 3; NLRP1/NLRP3: NOD-like receptors pyrin domain-containing 1 or 3; POM-1: polyoxotungstate-1.

## Abbreviations

AD: Alzheimer's disease
ALRs: AIM2-like receptors
ANSCs: Adult neural stem cells
APCs: Antigen-presenting cells
ASC: Apoptosis-associated speck-like protein containing a caspase recruitment domain
ATP: Adenosine triphosphate
CARD: Caspase recruitment domain
CB2R: Cannabinoid receptor 2
CNS: Central nervous system
CPZ: Chlorpromazine
CSF: Cerebrospinal fluid
DAMPs: Damage-associated molecular patterns
EAE: Experimental autoimmune encephalomyelitis
IAP: Integrin-associated protein
IFN*β*: Interferon-*β*
IL-1*β*: Interleukin-1*β*
mRNA: Messenger RNA
MS: Multiple sclerosis
NLRs: NOD-like receptors
NLRP3: NOD-like receptor pyrin domain-containing 3
NO: Nitric oxide
NOD: Nucleotide-binding oligomerization domain
PAMPs: Pathogen-associated molecular patterns
PD: Parkinson's disease
POM-1: Polyoxotungstate-1
PYD: Pyrin domain
Rac1-GTP: Rac1-guanosine triphosphate
ROS: Reactive oxygen species
ROT: Rotenone
RRMS: Relapsing-remitting multiple sclerosis
TLRs: Toll-like receptors
TNF: Tumor necrosis factor
TNFAIP3: Tumor necrosis factor *α*-induced protein 3
TRAF: TNF receptor-associated factor
SOCS: Suppressor of cytokine signaling.
